# A clinical approach to arrhythmias revisited in 2018: From ECG over noninvasive and invasive electrophysiology to advanced imaging

**DOI:** 10.1007/s12471-018-1089-1

**Published:** 2018-02-15

**Authors:** L. Jordaens

**Affiliations:** Department of Cardiology, University Hospital, Ghent, Belgium

**Keywords:** Arrhythmia mechanisms, Autonomic nervous system, Atrial fibrillation, Outflow tract arrhythmias, Ventricular extrasystoles

## Abstract

Understanding arrhythmias and their treatment is not always easy. The current straightforward approach with catheter ablation and device therapy is an amazing achievement, but does not make management of underlying or other cardiac disease and pharmacological therapy unnecessary. The goal of this paper is to describe how much of the knowledge of the 1980s and early 1990s can and should still be applied in the modern treatment of patients with arrhythmias. After an introduction, this review will focus on paroxysmal atrial fibrillation and a prototype of ‘idiopathic’ ventricular arrhythmias, two diseases with a striking similarity, and will discuss the arrhythmogenesis. The ECG continues to play an important role in diagnostics. Both diseases are associated with a structurally normal heart; the autonomic nervous system plays an important role in triggering arrhythmias at both the atrial and ventricular level.

## Introduction

**Fig. 1:**
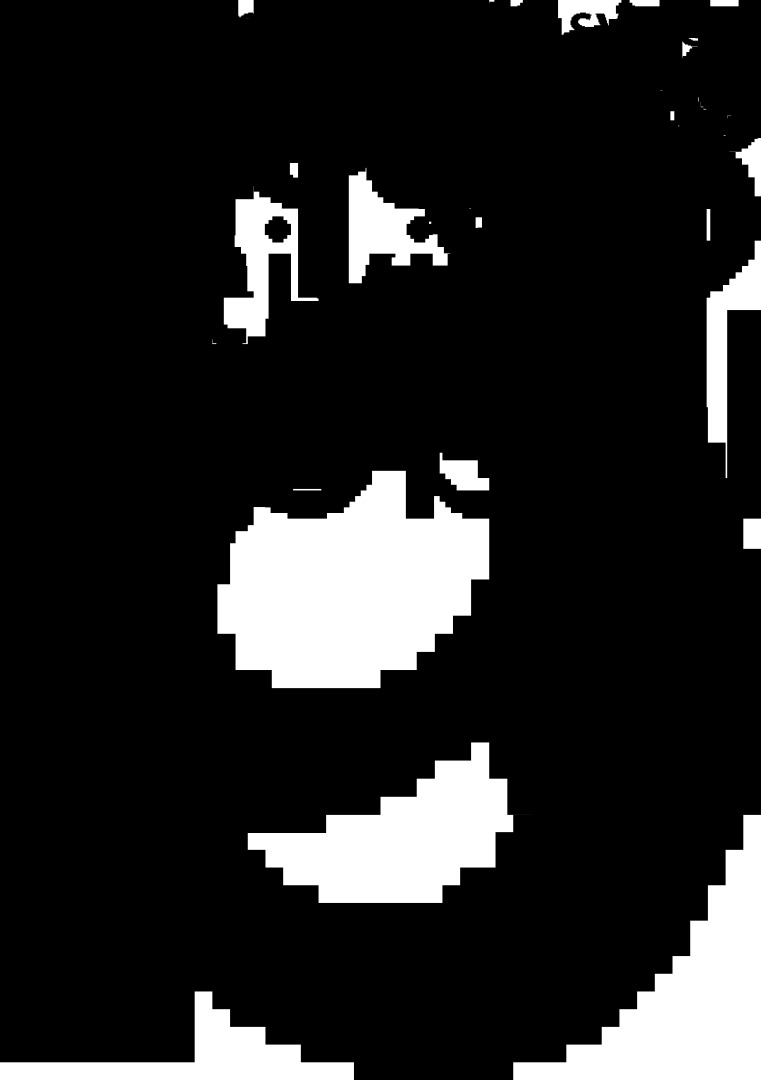
The original concept of Coumel on the left (**a**), and its generally well-known final form (**b**), as the triangle of Coumel, explaining how the interaction of substrate, triggers and the autonomic nervous system are important in arrhythmogenesis. (Modified after [2])

Recently, some concerns were expressed on the recent developments in arrhythmia management in general, and on the way clinical electrophysiology is evolving in particular [1]. This is a good reason to reemphasise some ideas on the clinical approach, and how this could help us in improving our understanding of atrial fibrillation (AF) and some important ventricular arrhythmias. This paper will review whether the very basic principles of the approach to arrhythmias (Fig. 1) as developed by Philippe Coumel can still be applied in the area of two frequently occurring arrhythmias, at the atrial and ventricular level, namely paroxysmal AF and ventricular extrasystoles of the outflow tract [2, 3]. These two prototypes were selected as they share some features, as similar changes in the substrate make the arrhythmia more cumbersome to treat.

## Conventional approach to arrhythmogenesis: normal impulses and arrhythmias

Understanding the genesis of arrhythmias (arrhythmogenesis) was not considered to be too difficult some 40 years ago, in the era before the advent of clinical electrophysiology with its ablation and implantable defibrillators. We only had the electrocardiogram at our disposal. Normal impulse generation in the sinus node and conduction through the specialised conduction tissue in the heart leads to electromechanical coupling, and the heart pumps the blood through the blood vessels. A calcium-mediated action potential governs normal impulse formation in the sinus node and in the atrioventricular (AV) node, while all other myocardial tissue is dependent on a sodium-channel mediated action potential. When this nice mechanism is disturbed, arrhythmias are encountered as the consequence of abnormal impulse generation or conduction, mainly due to re-entry [4, 5]. Automaticity, triggered activity, or bidirectional and unidirectional block were not considered too complicated to be part of the general medical curriculum. The work of Wellens and Durrer with programmed stimulation allowed us to study some of these mechanisms, especially re-entry, in depth [6].

## Conventional therapy (from the 1960s to early 1990s)

The widespread and well-taught pharmacological antiarrhythmic treatment at that time was only seldom subjected to randomised clinical trials [7]. You did not have to be a cardiologist to understand antiarrhythmic drug classification. However, for the more ambitious amongst us, a new scientific approach, the Sicilian Gambit, (like a move in a chess game), was proposed, the precursor of guidelines on this matter [8]. One should be able to identify the weak link of the rhythm disturbance, select the appropriate drug, and beat the arrhythmia. If this is impossible, some daring (and exceptional) antiarrhythmic surgery could be done in a few centres of excellence, both for atrial and ventricular arrhythmias [9, 10].

## Arrival of clinical electrophysiology (the late 1980s and the 1990s)

It was more or less at this moment that new options were added to our therapeutic arsenal. Class Ic drugs proved to be dangerous, at least for patients with ischaemic heart disease [11], but fortunately Michel Mirowski introduced the implantable cardioverter-defibrillator [12, 13]. Nephrologists warned me at that time that serious research on arrhythmias would be endangered, as there was no longer a need to understand, something they had observed with research on renal diseases after the arrival of dialysis. Luckily, this was not the case, as the almost simultaneous introduction of catheter ablation was a real boost for research on arrhythmias, and defibrillators proved to be nice ECG recorders, disclosing the arrhythmias involved in the genesis of sudden death [14–16]. A new subspeciality was born, clinical electrophysiology, and our generation was very glad to be part of it. In the long run, this magnificent technological revolution created a new generation of highly qualified cardiologists, the electrophysiologists, completely focussed on catheter ablation and device therapy (sometimes only on one of these aspects), but unfortunately, sometimes with limited knowledge on antiarrhythmic drugs, which now seemed less often necessary.

## Scientific advances of the last two decades

The insights into cardiac anatomy and functioning improved considerably. On the one hand, a growing basic knowledge, based on experimental animal studies, the development of molecular cardiology, and the fast evolution in genetics, improved our understanding of cardiomyopathies and diseases associated with arrhythmias [17, 18]. On the other hand, the developments in engineering, electro-anatomic mapping, and advances in imaging techniques as echocardiography and magnetic resonance proved to be extremely helpful in the diagnostics and therapy of bradyarrhythmias and tachyarrhythmias, and cardiology in general [19].

Antiarrhythmic treatment finally entered or re-entered the phase of randomised trials, often with disappointing results, so that over the last three decades only a few of the active antiarrhythmic drugs that were developed and investigated (e. g. azimilide, dronedarone and vernakalant) were actually put on the market, and then not always in all countries. This really did not help to improve the care of patients when catheter ablation or device therapy were unsuccessful, so, for instance with an incomplete effect of AF ablation or inappropriate shocks from implanted defibrillators. The real progress was not in the niche corner of a new antiarrhythmic, but rather with the advanced understanding of when and how to prescribe or avoid flecainide or amiodarone, with or without a beta-blocker, and how to treat background pathology and heart failure [20, 21].

## Electrocardiography versus imaging?

ECG interpretation remains important. This is not only the case for the 12-lead ECG, but also for Holter analysis, which lost much of its glory at the ventricular level after the CAST (Cardiac Arrhythmia Suppression Trial), as a matter of fact, without good reason [11]. Its use for AF detection is in my opinion not standardised, even if the guidelines and working parties for ablation have tried to make it so [22, 23]. This makes evaluation and understanding of AF interventions very difficult. A Holter is also a scientific tool providing information on the autonomic nervous system (ANS) [2]. Apart from the arrhythmia, mechanisms for arrhythmogenesis are hidden in the recording, and should be studied.

Imaging has evolved, with advanced body surface mapping, and processing of echocardiography and magnetic resonance. However, the real mechanism behind the arrhythmia is still not clarified. Moreover, the noninvasive technologies are not really assisting the general cardiologist, who has to make the first therapeutic decisions and also decide whether to transfer the patient to the electrophysiologist. His computer screen will finally display scars, barriers, rotors and wave fronts.

## Atrial arrhythmias: paroxysmal atrial fibrillation

AF is present in more than 20% of the population at a certain moment [24]. Other atrial arrhythmias all seem somehow related to AF: when not treated circus movement tachycardia, as in the Wolff-Parkinson–White syndrome in the young, often leads to AF at a higher age and there is an association between AVNRT, atrial flutter and AF [25]. Atrial extrasystoles predispose to AF. The breakthrough in understanding this disease is rather recent, with the discovery of spontaneous activity in the pulmonary veins [26]. One wonders how much of the hitherto described physiology remains valid, and how much is really applicable in the clinical situation. The focus will be on the patient with paroxysmal AF. Can the pathogenesis of AF be described in terms of triggers, autonomic activity and substrate, or is the presence of triggers enough to have AF?

### The trigger

Pulmonary veins are structures coated by muscular sleeves, which are remnants of the primitive heart, drawn into the lung tissue when the lungs are shaped during our early life. It is therefore not surprising that these muscular sleeves show spontaneous pacemaker activity, or that they form an electrical continuum with the adjacent atrium. Pulmonary vein isolation (PVI) has indeed become the cornerstone of effective interventional AF therapy [27]. The presence of multiple supraventricular extrasystoles on a Holter might be a good marker that PVI is the road to take. However, the pulmonary veins are not the only structures acting as a trigger—the superior vena cava, the coronary sinus, and Marshall’s ligament are all structures which can act as triggers, be it to a much lesser degree. The same can be said of the already-mentioned supraventricular arrhythmias.

### The substrate

Larger atria will more easily sustain AF than smaller ones, allowing multiple wavelets to re-enter [28]. The importance of this substrate is seen in the different outcome after PVI in paroxysmal versus persistent AF patients. In the latter form, the atria are larger, atrioventricular valves show more regurgitation, and more associated cardiovascular disease is seen. Even when these two forms might be the expression of a continuum, it is clear that all conditions stretching the atria (hypertension, valvular diseases, hyperthyroidism, excessive sports) will create inflammation, hypertrophy and fibrosis, probably preparing a setting to sustain re-entry when the triggers are active [29, 30]. This explains why all electrocardiographic and imaging indices showing larger atria or atrial overload (P-wave duration, longer signal averaged P waves, P‑wave dispersion, left atrial volume) will be helpful in predicting the outcome of cardioversion, drug therapy and PVI [31]. The same holds for the extent of atrial fibrosis as shown with magnetic resonance imaging, the holy grail of AF imaging [32]. Therefore, it is clear that careful assessment of the substrate before PVI is useful: a normal sized atrium is highly predictive for a successful procedure [27, 32].

### The ANS

The physiology of the autonomic innervation of the heart is intriguing. Bradycardia and tachycardia promote their own automaticity, not necessarily related to the ANS [33]. The sinus node, the atria and the AV node are controlled by the vagal and adrenergic nervous system. Apart from its effects on heart rate, the ANS contributes to inhomogeneous activation of the atria, and has effects on conduction and repolarisation creating or maintaining the arrhythmia. Coumel made a very clear and didactic distinction between vagally induced and adrenergic mediated forms of AF [16]; the first occurred in the setting of a normal heart, at rest, while the second typically occurred during exercise, and suggested the presence of a more pathological and damaged substrate. The first type of AF was not to be treated with beta-blockers, while this was considered to be the perfect therapy for the second type. From a clinical point of view, only a minority of AF patients seem to correspond to these prototypes, and many are mixed [34]. Is the role of the ANS then only marginal? In specific situations, the influence of the ANS can be more pronounced, as in postoperative situations, where vagal withdrawal, with sudden adrenergic changes, can provoke AF [35]. Epicardial fat pad ablation was found to be successful to prevent AF after coronary bypass [36]. According to some, PVI should be completed by additional ablation of the autonomic ganglia, while a recent surgical trial was negative [37]. It cannot be excluded that the success of large circumferential ablation (as opposed to segmental PVI) is due to modulation of the antra, where many of these structures are situated [38]. Furthermore, beta-blocking agents are the only drugs withstanding the scrutiny of the Cochrane review of antiarrhythmic drugs, with a significant reduction of AF recurrence, and a low incidence of side effects [39]. Therefore, I feel that the influence of the ANS should still be assessed in all patients, even when this is limited to a simple Holter recording.

## Ventricular arrhythmias: the outflow tract

Sudden cardiac death is very often the end result of an interaction of the three factors in the triangle of Coumel [22]. Risk stratification, which was extremely popular in the late 1990s, was largely neglected after the observation that a low left ventricular ejection fraction (LVEF) was the more important parameter, but has now been taken up again as it becomes clear that the old flowcharts are not so good in modern times as expected, e. g. for non-ischaemic cardiomyopathy [40, 41]. The role of the ANS becomes increasingly clear when the impact of beta-blockers is considered on event-free survival, for example in heart failure [42]. Neuromodulation (left-sided stellectomy, thoracic epidural anaesthesia) is being studied, especially in patients at high risk [43].

This review will now focus on a particular condition in the normal heart, first described by Gallavardin, and also well studied by Coumel, namely extrasystoles originating in the outflow tract. When occurring as tachycardia, it was called ‘tachycardie en salves’, and more recently ‘repetitive monomorphic tachycardia’ [44].

**Fig. 2:**
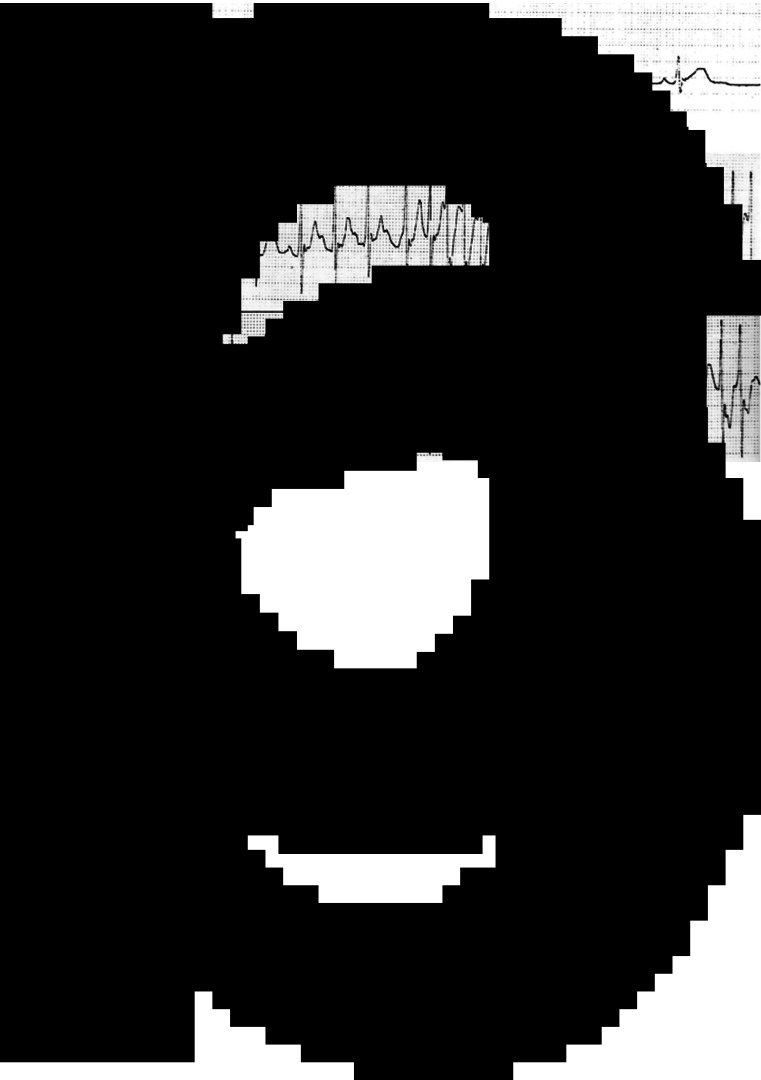
Holter recording of a professional cyclist with symptomatic atrial fibrillation and dizziness. **a** Bradycardia at rest, with a short episode of slow conduction of atrial flutter or fibrillation. **b** During exercise atrial flutter, with suddenly 1:1 conduction. **c** Continuation of **b**, with slowing of the atrioventricular conduction at the end

This variant of ventricular extrasystoles or tachycardia is almost ubiquitous, with a typical left bundle branch morphology and inferior axis [45]. The reason for the omnipresence of this kind of extrasystole is the fact that the outflow tract of the primitive embryonic heart (the venous sinus horn) serves as a pacemaker, and retains the capacity to generate electrical activity in later life, in a different way than the other myocardial cells [46]. The sinus node should take the lead, and suppress its activity. It might be postulated that in the presence of other factors, these extrasystoles become important again and might even lead to sustained tachycardia (Fig. 2), and to tachycardiomyopathy [47].

### The trigger

Whether these extrasystoles are due to abnormal calcium currents or not, or to delayed activity with afterdepolarisation or enhanced automaticity is important when we are looking for a medical therapy [48]. Adenosine has an important diagnostic role, and calcium antagonists may be effective in suppressing extrasystoles [49]. It is not clear in clinical practice whether the latter is due to a direct activity on the action potential, or whether the result is due to an effect on the heart rate. There is no reason to believe (in contrast to what is published in the guidelines) that outflow tract arrhythmias from the left or right side would behave in a different way [50]. The ECG plays an important role in diagnostics, for localisation of the origin, and the Holter discloses the burden of the arrhythmia, which is important for decision-making [45, 47].

### The substrate

Right ventricular outflow tract arrhythmias are considered benign (and were also called benign ventricular tachycardia), indicating that the myocardium is considered to be healthy [51]. This means that no structural heart disease should be detected, that the ECG shows no signs of the Brugada syndrome, that no signs of arrhythmogenic heart disease are present, and so on. Exercise testing shows no signs of ischaemia. Valvular disease should be absent, but the extrasystoles might increase or provoke insufficiency, creating a difficult to interpret haemodynamic perturbation during echocardiography. Nevertheless, it is clear that this kind of extrasystoles might coexist with a pathological heart. Extrasystoles from the outflow tract may provoke ventricular fibrillation or tachycardia in coexistent ischaemic heart disease (even during acute infarction), dilated cardiomyopathy, Brugada syndrome, congenital heart disease, and in arrhythmic right ventricular cardiomyopathy (ARVC) [52, 53].

Abnormalities on magnetic resonance and echocardiography are only seldom detected, and with the present technology can be considered anecdotal. Nevertheless, it is hypothesised that many athletes seen with outflow tract tachycardia have developed fibrosis over time, making re-entry possible [54]. This fibrosis is reflected in a longer QRS duration, and impacts on repolarisation [55, 56]. It is unlikely that all the athletes in our study had ARVC, or hypertrophic cardiomyopathy, as further invasive and noninvasive studies did not show clues for additional pathology.

### The ANS

**Fig. 3:**
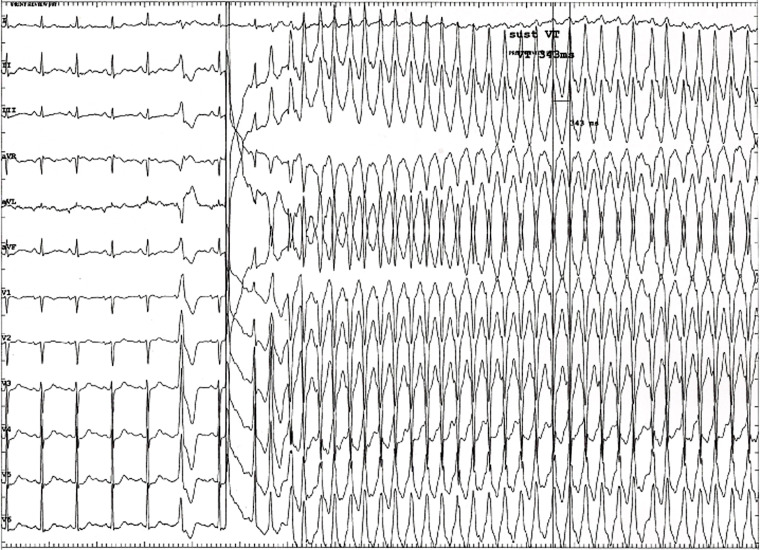
Drug study of a marathon runner with syncope. The 6th complex shows a ventricular extrasystole, negative in lead I, and positive in the inferior leads. The transition is very early, indicating a left ventricular origin. After isuprel infusion, a fast sustained tachycardia occurs, initiated by a similar left ventricular complex, while the VT suggests a right ventricular outflow tract origin

Neurohumoral factors play an important role in the occurrence of arrhythmias and in the development of symptoms. Extrasystoles appear typically at a certain heart rate, and disappear at higher frequencies. A close relation was detected between the heart rate and duration of ventricular runs, and even with the coupling interval of the first extrasystole [57]. Exercise testing often results in more, rather than in less arrhythmias, which is to be considered abnormal. Infusion of isoprenaline may provoke the arrhythmia spontaneously (Fig. 3), or facilitate inducibility, which could suggest support for triggered activity and re-entry as a mechanism. More advanced Holter analysis shows that altered dynamics of the heart rate, as caused by sympathetic activation, may precondition the heart to ventricular fibrillation [58].

The ESC guidelines give no real clues for reliable drug therapy, but there is agreement that medical therapy is disappointing [59]. Beta-blocking agents also yield disappointing results. It might be that the endpoint set for successful therapy is wrong: beta-blockers probably do not erase the arrhythmia (i. e. the trigger) but they may still be capable of preventing some complications, and play a role in the prevention of heart failure [42]. Many patients with outflow tract arrhythmias also suffer from other arrhythmias, often heavily influenced by adrenergic tone: AV-nodal re-entry and AF [60]. The advantages of beta-blockade may outweigh the disadvantages of the class Ic agents, which are advocated in the guidelines. They may also be necessary after catheter ablation.

## Conclusion

**Fig. 4:**
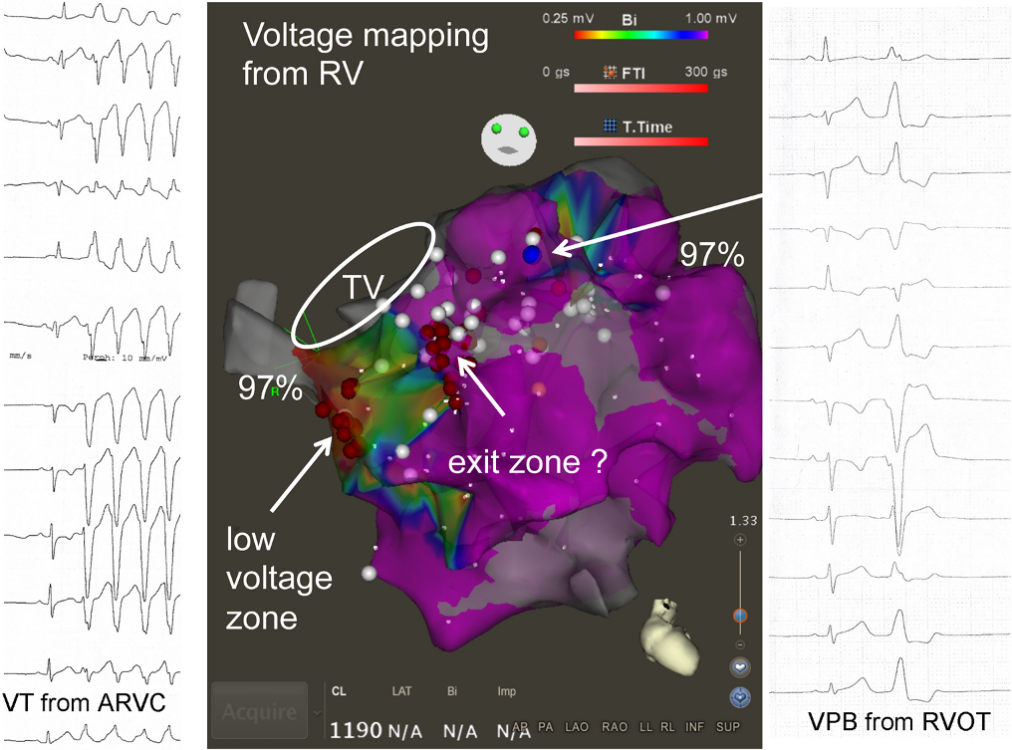
Coexistent ventricular tachycardia (*VT*) in a patient with arrhythmogenic right ventricular cardiomyopathy (*ARVC*), and ventricular extrasystoles (*VPB*) originating in the right ventricular outflow tract (*RVOT*)

It is concluded that for the daily, clinical, but also for the advanced interventional management of patients a consideration of the classical three mechanisms of arrhythmogenesis remains useful. Both electrocardiography and imaging (Fig. 4) remain important to come to a correct diagnosis in both the examples discussed here, knowing that imaging will not reveal many abnormalities at first glance. The autonomic nervous system plays a role in both conditions, and its importance is sometimes difficult to quantify.

Further, the parallelism of arrhythmogenesis in atrial and ventricular arrhythmias as described in this selective review is striking. Attempts to prevent atrial and ventricular overloading, and fibrosis (if possible) are of the highest importance. Beta-blockers remain safer than other antiarrhythmic drugs and may prevent benign arrhythmias from becoming malignant.

Finally, clinical electrophysiology provides us with important tools to improve this conventional approach. More refined mapping is necessary, and devices are magnificent recorders. We will need electrophysiology specialists who know a lot about the basics and other areas of cardiology to make progress.
